# Botulinum Toxin Type A Induces Changes in the Chemical Coding of Substance P-Immunoreactive Dorsal Root Ganglia Sensory Neurons Supplying the Porcine Urinary Bladder

**DOI:** 10.3390/toxins7114797

**Published:** 2015-11-16

**Authors:** Agnieszka Bossowska, Ewa Lepiarczyk, Urszula Mazur, Paweł Janikiewicz, Włodzimierz Markiewicz

**Affiliations:** 1 Department of Human Physiology, Faculty of Medical Sciences, University of Warmia and Mazury in Olsztyn, Warszawska 30, Olsztyn 10-082, Poland; E-Mails: ewa.lepiarczyk@uwm.edu.pl (E.L.); urszula.maslowska@uwm.edu.pl (U.M.); pawel.janikiewicz@student.uwm.edu.pl (P.J.); 2 Department of Pharmacology and Toxicology, Faculty of Veterinary Medicine, University of Warmia and Mazury in Olsztyn, Oczapowskiego 13, Olsztyn 10-719, Poland; E-Mail: mark@uwm.edu.pl

**Keywords:** botulinum toxin A, urinary bladder, sensory innervation, dorsal root ganglia, substance P, neurotransmitters, pain, immunohistochemistry, pig

## Abstract

Botulinum toxin (BTX) is a potent neurotoxin which blocks acetylcholine release from nerve terminals, and therefore leads to cessation of somatic motor and/or parasympathetic transmission. Recently it has been found that BTX also interferes with sensory transmission, thus, the present study was aimed at investigating the neurochemical characterization of substance P-immunoreactive (SP-IR) bladder-projecting sensory neurons (BPSN) after the toxin treatment. Investigated neurons were visualized with retrograde tracing method and their chemical profile was disclosed with double-labelling immunohistochemistry using antibodies against SP, calcitonin gene-related peptide (CGRP), pituitary adenylate cyclase activating polypeptide (PACAP), neuronal nitric oxide synthase (nNOS), galanin (GAL), calbindin (CB), and somatostatin (SOM). In the control group (*n* = 6), 45% of the total population of BPSN were SP-IR. Nearly half of these neurons co-expressed PACAP or CGRP (45% and 35%, respectively), while co-localization of SP with GAL, nNOS, SOM or CB was found less frequently (3.7%, 1.8%, 1.2%, and 0.7%, respectively). In BTX-treated pigs (*n* = 6), toxin-injections caused a decrease in the number of SP-IR cells containing CGRP, SOM or CB (16.2%, 0.5%, and 0%, respectively) and a distinct increase in these nerve cells immunopositive to GAL (27.2%). The present study demonstrates that BTX significantly modifies the chemical phenotypes of SP-IR BPSN.

## 1. Introduction

Botulinum toxin (BTX), produced by anaerobic bacteria Clostridium botulinum [1] is one of the most potent neurotoxins known. It acts by entering the presynaptic bulb of cholinergic neurons and preventing acetylcholine (ACh) release into the synaptic cleft [2]. Once internalized by nerve terminals, BTX cleaves specific sites of synaptosome-associated protein 25 (SNAP-25), and therefore inhibits the soluble *N*-methylmaleimide-sensitive attachment protein receptor (SNARE)-mediated fusion of synaptic vesicles with the neuronal membrane [3]. The inhibitory influence exerted by BTX on both the somatic and autonomic innervation is well-known. It has been shown that this toxin reduces the release of ACh at the neuromuscular junction [4] or from efferent nerve terminals in the lower urinary tract [5]. Because BTX administration results in suppressing muscle contractility, so far, the toxin has been proposed for the treatment of several neurogenic dysfunctions, characterized by excessive or inappropriate muscle contractions, such as strabismus, blepharospasm or muscular dystonias (for review, see [6,7]).

Apart from the well-known influence of BTX on the somatic and cholinergic innervation, there is a growing body of evidence that this toxin may also inhibit afferent neurotransmission and has analgesic properties in animals and humans. Filippi and co-workers [8] showed that local injections of BTX in the deep masseter muscle in rats directly reduce afferent 1a fiber activation and thereby produce a modulatory effect on sensory feedback. It was demonstrated that BTX inhibits the release of substance P (SP) [9] and calcitonin gene-related peptide (CGRP) [10,11,12], which are involved in the genesis of pain, from rat cultured embryonic dorsal root ganglia (DRG) and trigeminal ganglion neurons, respectively. Moreover, in an animal model of inflammatory pain, it was shown that BTX may reduce a formalin-induced release of glutamate [13] (glutamate is a factor responsible for stimulating local nociceptive neurons through activation of receptors located on peripheral afferents [14]). In humans, Jabbari and collaborators [15] reported that segmental burning pain in patients suffering from spinal cord lesions was relieved by multiple subcutaneous injections of BTX.

Application of BTX in the treatment of lower urinary tract symptoms (LUTS) has expanded greatly in the recent years. Injections of BTX have successfully been used to relieve the urological conditions such as refractory detrusor hyperreflexia [16], detrusor sphincter dyssynergia [17], overactive bladder syndrome [18], neurogenic bladder overactivity [19,20,21] or interstitial cystitis/painful bladder syndrome [22,23].

Because of its mechanism of action, BTX was suspected to selectively influence urinary bladder cholinergic neurons. It has been shown that this neurotoxin inhibits ACh release from efferent nerve terminals in the lower urinary tract [5]. Furthermore, Lepiarczyk *et al.* [24] have revealed that BTX causes profound changes in the pattern of distribution, relative frequency, and chemical coding of cholinergic nerve fibers supplying the porcine urinary bladder wall. However, recently published data clearly suggest that BTX may also significantly influence the sensory innervation of the lower urinary tract tissues. It has been proven, for example, that this toxin inhibits afferent nerve-mediated bladder strip contractions [25]. The mechanism of BTX action on the sensory innervation of the bladder is probably associated with inhibiting the release of neurotransmitters such as SP and CGRP from peripheral afferent nerve terminals projecting to this organ [26,27]. It has also been suggested that this toxin may cause a reduction of sensory fibers immunoreactive to P2X3 and transient receptor potential channel, vanilloid family member 1 (TRPV1) receptors in the bladder [28] therefore leading to the inhibition of the protein kinase C (PKC) mediated SNARE-dependent exocytosis of TRPV1 to the plasma membrane [29]. Moreover, Bossowska and Majewski [30] revealed that in pigs, BTX intravesical injections result in significant changes in the chemical coding of DRG sensory neurons. These findings strongly suggest that BTX influences not only the parasympathetic, but also the sensory subdivision of the bladder innervation.

The analysis of available literature clearly reveals that SP, which is often considered as a marker of sensory neurons, performs several different functions in the urinary bladder tissues. For instance, SP is involved in the mechanoreceptor-mediated micturition reflex [31,32,33], urinary bladder hyperreflexia [34] and pain transmission from this organ [35]. It has also been documented, that this neuropeptide is involved in triggering several inflammatory responses [36].

Interestingly, the results of our previous study [30] reveal that BTX treatment distinctly decreases the number of bladder-projecting sensory neurons (BPSN) containing SP. Therefore, also taking into consideration multidirectional regulatory action of SP in the lower urinary tract, the present study was designed to investigate the chemical coding of DRG SP-IR sensory neurons projecting to urinary bladder in normal (intact) pigs and in BTX-treated animals. The experiment was performed using the combined retrograde tracing method and double-labelling immunohistochemistry. As was mentioned before, nowadays BTX is used in the treatment of various urinary bladder disorders in humans. Because it has been found that these disorders are more common in women compared with men [37], we decided to examine female animals in the present research. We used domestic pigs as an experimental animal model, as they share similar anatomical and physiological features with humans with respect to many organ systems, including the urinary tract (e.g., [38,39,40]).

## 2. Results and Discussion

### 2.1. Distribution of SP-Containing BPSN Neurons in Control and BTX-Treated Animals

In our previous study [30,41] it had already been established that in the control animals Fast Blue-positive (FB+) BPSN immunopositive to SP (SP+) accounted for the largest population (45.2% ± 14.07%; mean ± standard deviation—SD) among all the retrogradely labeled sensory nerve cells. It had also been determined, that BTX-injections have a significant impact on SP+ BPSN, as they resulted in a substantial decrease of neurons belonging to this subpopulation (19.3% ± 5.1%). Therefore, as it seems that FB+/SP+ BPSN have the greatest contribution in the sensory innervation of the urinary bladder wall in the pig, the purpose of the present experiment was to investigate in detail the chemical coding of these neurons. Nevertheless, it should be emphasized that the number, sex, body weight, and age of the animals used in the present experiment and in our previous studies [30,41] as well as the surgical and immunohistochemical procedures applied were entirely corresponding.

With regard to the diameter of SP+ BPSN, in both the control and BTX-treated animals, only two classes of these nerve cells have been determined. The vast majority of FB+/SP+ neurons found in all the DRG studied in both experimental groups included small-sized cells (average diameter up to 30 µm; 83.1% ± 4.5% *vs.* 82.8% ± 2.1%). The bladder sensory SP-positive medium-sized neurons (diameter 31–50 µm) were less numerous (16.9% ± 3.9% *vs.* 17.2% ± 2.8%). The large BPSN (diameter >50 µm) containing SP were not found in the DRG studied.

**Scheme 1:**
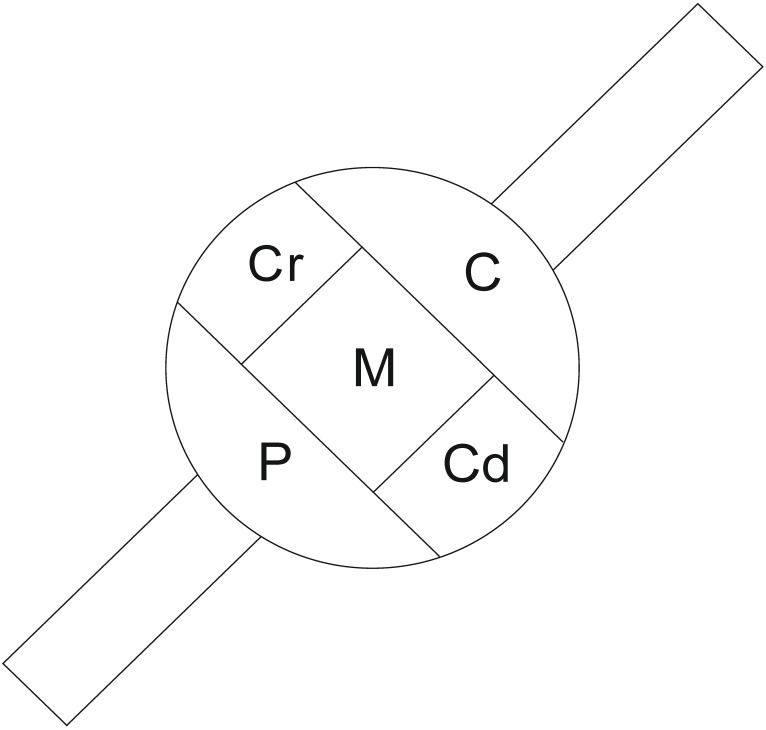
A schematic diagram of a spinal ganglion section showing its arbitrary division into topographical domains, in which the occurrence and relative frequency of substance P (SP) containing bladder projecting sensory neurons (BPSN) were studied. Presented data were pooled from all dorsal root ganglia (DRG) sections studied, both the ipsi- and contralateral ganglia. **C**—central, **P**—peripheral, **Cr**—cranial and **Cd**—caudal domains of the DRG, **M**—middle region of the ganglion.

In both the control group and BTX-treated pigs, the majority of FB+/SP+ neurons was unevenly distributed, as isolated cells scattered throughout the individual ganglia. Only few SP+ BPSN formed small, loose clusters up to three neurons. In both experimental groups FB+/SP+ neurons were present in different domains of sensory ganglia. A distinct accumulation of the retrogradely labelled SP-positive cells was found in the caudal (31.4% ± 1.8% *vs.* 30.9% ± 3.2%) and in the cranial part (29.8% ± 1.0% *vs.* 29.6% ± 1.2%) of DRG studied. Definitely, a lower number of SP+ sensory neurons supplying the urinary bladder was observed in the central, middle, and peripheral part of the ganglia (Scheme 1; 15.2% ± 0.7% *vs.* 14.3% ± 2.2%, 13.1% ± 1.0% *vs.* 12.9% ± 0.7% and 10.5% ± 1.0% *vs.* 12.3% ± 2.4%, respectively).

### 2.2. Immunohistochemical Characteristic of SP-Containing BPSN Neurons in Control and BTX-Treated Animals

The technique of double immunohistochemical staining revealed two main subpopulations of FB+/SP+ neurons in the control group as well as in the BTX-treated animals. The majority (63.6% ± 3.0% *vs.* 70.8% ± 6.5%) of the retrogradely labeled SP-positive sensory cells revealed also immunoreactivity to other investigated biologically active substances. The remaining FB+ neurons (36.4% ± 3.0% *vs.* 29.2% ± 6.5%) were immunopositive only to SP.

**Figure 1:**
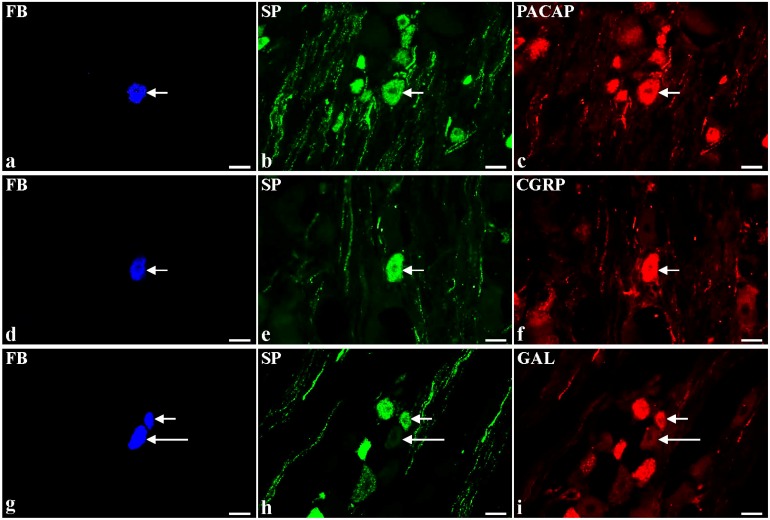

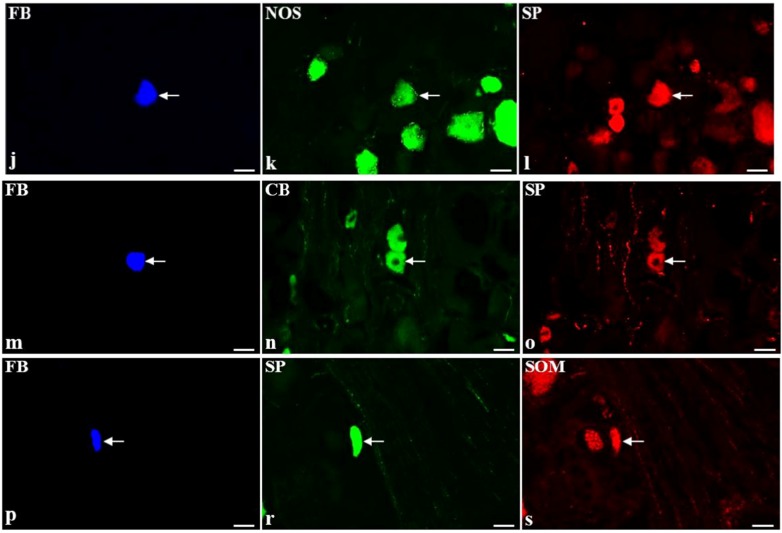
Representative images of substance P-positive (SP+) dorsal root ganglia (DRG)-urinary bladder-projecting neurons (UBPN) in control pigs. All images were taken separately from blue (**a**,**d**,**g**,**j**,**m**,**p**), green (**b**,**e**,**h**,**k**,**n**,**r**) and red (**c**,**f**,**i**,**l**,**o**,**s**) fluorescent channels; **a–c** One fast blue-positive (FB+) neuron (**a**, blue, arrow), which simultaneously contains SP (**b**, green, arrow) and pituitary adenylate cyclase activating peptide-PACAP (**c**, red, arrow). **d–f** One FB+ neuron (**d**, blue, arrow), which is simultaneously SP+ (**e**, green, arrow) and calcitonin gene-related peptide-positive (CGRP+, **f**, red, arrow). **g–i** Two FB+ neurons (**g**, blue, 1 short arrow, 1 long arrow), which are simultaneously SP+ (**h**, green, 1 short arrow) or SP-negative (**h**, green, 1 long arrow) and galanin-positive (GAL+, **i**, red, 1 short arrow) or GAL-negative (GAL-, **i**, red, 1 red arrow). **j–l** One FB+ neuron (**j**, blue, arrow), which simultaneously contains neuronal nitric oxide synthase-nNOS (**k**, green, arrow) and SP (**l**, red, arrow). **m–o** One FB+ neuron (**m**, blue, arrow), which is simultaneously calbindin-positive (CB+, **n**, green, arrow) and SP+ (**o**, red, arrow). **p-s** One FB+ neuron (**p**, blue, arrow), which simultaneously contains SP (**r**, green, arrow) and somatostatin-SOM (**s**, red, arrow). Bars 50 μm (**a**–**s**).

In the control pigs, almost half of the FB+/SP+ neurons additionally contained pituitary adenylate cyclase activating peptide (PACAP, Figure 1a–c; 45.1% ± 1.3%). Many of the SP-positive BPSN revealed also immunoreactivity to CGRP (Figure 1d–f; 35.6% ± 8.2%). A small number of these neurons (3.7% ± 0.3%) stained for galanin (GAL; Figure 1g–i) but only single FB+/SP+ neuronal somata were neuronal nitric oxide synthase (nNOS; Figure 1j–l)-, somatostatin (SOM; Figure 1p–s)- or calbindin (CB; Figure 1 m–o)-immunopositive (1.8% ± 0.6%, 1.2% ± 0.7% and 0.7% ± 0.7%, respectively).

**Figure 2:**
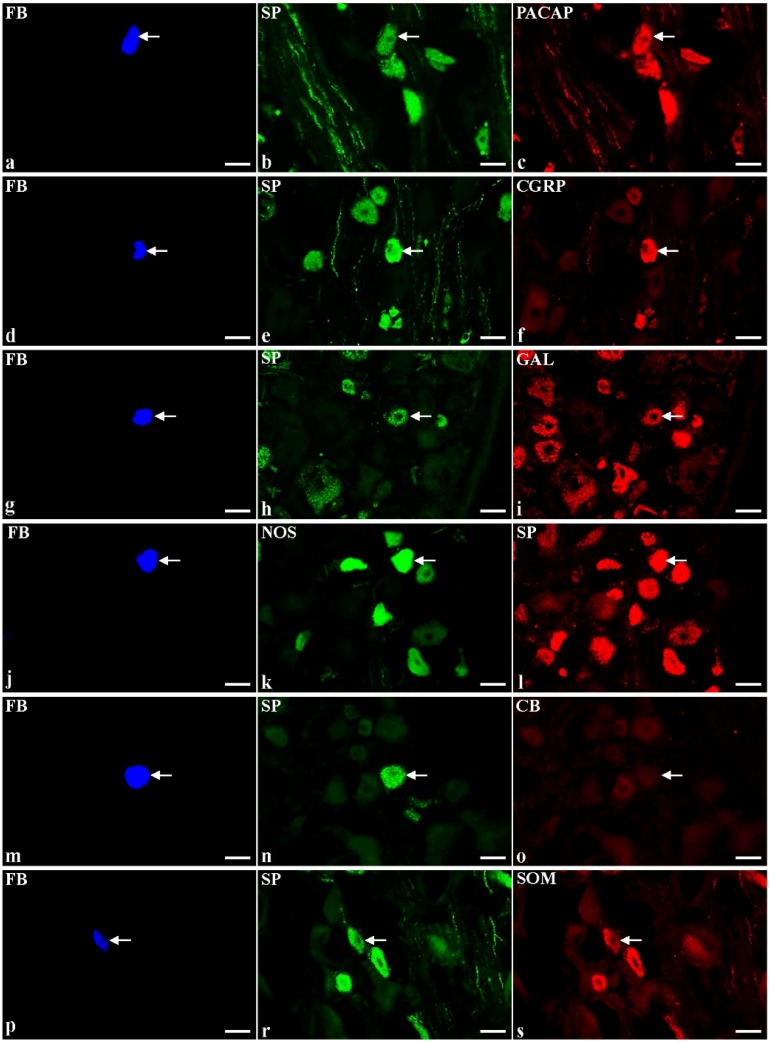
Representative images of substance P-positive (SP+) dorsal root ganglia (DRG)-urinary bladder-projecting neurons (UBPN) in BTX-treated animals. All images were taken separately from blue (**a**,**d**,**g**,**j**,**m**,**p**), green (**b**,**e**,**h**,**k**,**n**,**r**) and red (**c**,**f**,**i**,**l**,**o**,**s**) fluorescent channels; **a–c** one fast blue-positive (FB+) neuron (**a**, blue, arrow), which simultaneously contains SP (**b**, green, arrow) and pituitary adenylate cyclase activating peptide-PACAP (**c**, red, arrow). **d–f** One FB+ neuron (**d**, blue, arrow), which is simultaneously SP+ (**e**, green, arrow) and calcitonin gene-related peptide-positive (CGRP+, **f**, red, arrow). **g–i** One FB+ neuron (**g**, blue, arrow), which is simultaneously SP+ (**h**, green, arrow) and galanin-positive (GAL+, **i**, red, arrow). **j–l** One FB+ neuron (**j**, blue, arrow), which simultaneously contains neuronal nitric oxide synthase-nNOS (**k**, green, arrow) and SP (**l**, red, arrow). **m–o** One FB+ neuron (**m**, blue, arrow), which is simultaneously SP+ (**n**, green, arrow) and calbindin-negative (CB-, **o**, red, arrow). **p-s** One FB+ neuron (**p**, blue, arrow), which simultaneously contains SP (**r**, green, arrow) and somatostatin-SOM (**s**, red, arrow). Bars 50 μm (**a**–**s**).

BTX-injections led to a significant decrease in the number of retrogradely labelled SP-IR cells simultaneously containing CGRP (Figure 2d–f; 16.2% ± 3.8%), SOM (Figure 2p–s; 0.5% ± 0.6%) or CB (Figure 2m–o; 0%) and caused a distinct increase in the SP- and GAL-IR BPSN (Figure 2g–i; 27.2% ± 0.4%). Furthermore, there were no significant differences in the number of FB+/SP+ sensory neurons expressing immunoreactivity to PACAP (Figure 2a–c; 45.7% ± 6.0%) or nNOS (Figure 2j–l; 1.8% ± 2.5%).

The fluorescence colocalization study of SP with other neuropeptides (or their markers) performed on consecutive sections of FB+ BPSN neurons allowed to observe the coexistence of more than one biologically active substance in the same SP-positive neuron, both in the control and BTX-treated group. Generally the largest population of FB+/SP+ BPSN coexpressed also PACAP and CGRP. What is more, some of the neurons belonging to the mentioned two main subpopulations of SP+ BPSN additionally revealed immunoreactivity to GAL, nNOS, SOM and/or CB. In the group of BTX-treated pigs, the subpopulation of SP+/PACAP+ retrogradely labelled neurons mainly contained GAL and/or CGRP but only a small number of these cells were simultaneously immunopositive to nNOS. Details concerning the relative percentages of individual subpopulations of retrogradely labeled SP+ neurons in both the control group and in the group of BTX-treated animals are shown in Table 1.

**Table 1:** The percentages of retrogradely labelled substance P (SP)-containing neuronal subpopulations in the dorsal root ganglia (DRG) in the control pigs and in the animals after botulinum toxin (BTX) treatment (calcitonin gene-related peptide—CGRP, pituitary adenylate cyclase activating polypeptide—PACAP, galanin—GAL, neuronal nitric oxide synthase—nNOS, somatostatin—SOM, calbindin—CB). Data expressed as mean ± standard deviation (SD). Asterisks mark statistically significant differences at ** *p* <0.001.

Collocation Patterns of SP with Different Neurotransmitters in the Bladder DRG Neurons	The Group of Control Animals	The Group of BTX-Treated Animals
SP+/CGRP+/PACAP+	11.0% ± 5.1%	10.9% ± 1.3%
SP+/PACAP+/GAL+	0.8% ± 0.4%	**13.3% ± 3.5%
SP+/PACAP+/nNOS+	0.7% ± 0.2%	1.2% ± 0.4%
SP+/CGRP+/GAL+	0.5% ± 0.5%	0%
SP+/CGRP+/SOM+	0.4% ± 0.4%	0%
SP+/PACAP+/CB+	0.3% ± 0.4%	0%
SP+/CGRP+/PACAP+/GAL+	1.6% ± 0.9%	3.1% ± 0.7%
SP+/PACAP+/GAL+/nNOS+	0.4% ± 0.3%	0%
SP+/CGRP+/PACAP+/nNOS+	0.2% ± 0.2%	0%
SP+/CGRP+/PACAP+/SOM+/GAL+	0.5% ± 0.4%	0.6% ± 0.3%
SP+/CGRP+/PACAP+/SOM+/GAL+/CB+	0.3% ± 0.3%	0%

## 3. Discussion

The results of the present experiment confirm that DRG is an important source of the sensory innervation of the urinary bladder in the female pig. A comprehensive discussion dealing with the distribution of BPSN in DRG has already been presented in our previous paper [41]. It should be stressed that the number, sex, body weight, and age of the animals used as controls in both experiments as well as the surgical and immunohistochemical procedures applied were entirely corresponding. In the present study, we additionally examined the distribution of the investigated nerve cells in the individual ganglia considering their different domains described in Scheme 1. We found that DRG BPSN were unevenly distributed throughout the individual ganglia but with the highest concentration in their caudal and cranial parts. It is hard to compare these observations with respect to data provided by other authors, because investigations dealing with, for instance, detailed distribution of the BPSN in DRG have not been performed so far. However, Russo *et al.* [42] revealed that in the boar the bladder trigone-innervating sensory neurons were mainly distributed at the proximal pole of the DRG. The dissimilarities observed between male and female pigs may probably reflect the sex-dependent variations or may be a consequence of different locations of FB injection sites chosen in the researches.

As was already mentioned in the introduction section, in our previous study [41] it has been found that the largest population of DRG BPSN expressed immunoreactivity to SP. Moreover, it was established earlier that BTX treatment causes a distinct decrease in the number of these nerve cells [30]. The results of the present experiment suggest, however, that the function of SP may be regulated/modified by many other neurotransmitters which simultaneously to SP are localized in the same DRG BPSN.

The results obtained in the present experiment indicate that BTX can be considered as a factor evoking distinct adaptation changes in SP+ DRG BPSN. These changes include a decrease in the number of SP+ BPSN containing simultaneously CGRP, SOM, or CB and an increase in the number of SP+ perikarya immunopositive to GAL. There are several possible mechanisms explaining this phenomenon. Firstly, the changes observed may result from reduction of the peripheral formation and retrograde uptake of trophic molecules after BTX treatment [43]. It is well known, for instance, that neurotrophic growth factor (NGF) increases SP and CGRP synthesis in DRG [44], therefore inhibition of NGF production should be followed by a decrease in the number of SP- and CGRP-IR bladder-projecting sensory neurons in the BTX-injected animals. Apart from NGF, glial cell line-derived neurotrophic factor (GDNF) is also considered as an agent responsible for proper neurotransmitter secretion. It was shown, for instance, that prolonged intrathecal treatment with GDNF increased the number of SOM-containing sensory neurons in DRG and the actively induced release of SOM in the dorsal horn isolated *ex vivo* [45]. Because it has been found that BTX leads to a reduction in NGF production in the human bladder [43], it is possible that this neurotoxin also inhibits NGF, and perhaps GDNF, production in the porcine bladder tissue, as well, which in turn, may cause a decrease in SP, CGRP or SOM expression in bladder-projecting sensory neurons. This mechanism may also be true for other neurotransmitters investigated in the present experiment.

Another possible explanation of the observed changes in the immunohistochemical properties of SP+ DRG BPSN is that peripherally injected BTX moves along the peripheral nerves via retrograde axonal transport thus reaching DRG in which the toxin protease inhibits neurotransmission by SNAP-25 cleavage [46,47]. Papagiannopoulou *et al.* [48] revealed in rats, that injections of the urinary bladder with BTX were followed by a significant accumulation of the toxin in the lumbosacral DRG. This, consequently, may give rise to changes in the secretion of certain neurotransmitters to the synaptic cleft.

Furthermore, the changes in the immunohistochemical properties of the investigated neurons may result from reaction of the peripheral nerves to inflammation caused by the toxin administration process. Nevertheless, is should be stressed that the urinary bladder wall was carefully examined before collecting the DRG, and no evident macroscopic and microscopic signs suggesting an inflammatory reaction were found. This finding is not surprising, considering the fact that BTX exerts an anti-inflammatory effect on the tissues injected [49].

The present investigation has revealed that almost half of the SP-IR FB+ sensory neurons were simultaneously immunopositive to PACAP. Moreover, it was found that BTX injections did not change the number of these nerve cells.

It is difficult to discuss the possible impact of PACAP on the function of SP in the DRG BPSN, as the physiological roles of these two neurotransmitters in the urinary tract tissues are usually described separately. It has been suggested that under physiological conditions, SP released from bladder sensory neurons participates in the mechanoreceptor-mediated micturition reflex. For instance in rats, systemic administration of capsaicin for reduction of SP, was followed by the retention of urine or by increased volume/pressure threshold for micturition, suggesting that SP has an excitatory function in the afferent micturition pathway [32]. It has also been demonstrated that SP released from the central nerve terminals of capsaicin-sensitive bladder afferent neurons may be involved in mediating urinary bladder hyperreflexia [34,50]. Moreover, it has been found that noxious peripheral stimuli may elicit SP release from the central endings of DRG perikarya [35]. Because these endings are known to synapse on the dorsal part of the sacral parasympathetic nucleus [33], it has been assumed that SP may play an excitatory role in many types of bladder reflexes in rat [31]. Moreover, in the urinary tract tissues SP is thought to be responsible for transmission of nociceptive information [50] and for triggering the inflammatory responses [36].

Several possible mechanisms of PACAP physiological functions in the lower urinary tract have been suggested. It has been found that this neurotransmitter plays an important role in facilitation of the urinary bladder smooth muscle contractions in rats [51,52]. Moreover, Girard and collaborators [53] suggested that PACAP signaling acts as an agent in the regulation of bladder contractility at the level of the bladder urothelium. It has been also observed that PACAP mediates nociceptive signaling in mice [54,55] and is involved in the genesis of the urinary bladder inflammation process in rats [56,57]. Furthermore, upregulation of PACAP in the urinary bladder tissues was demonstrated after chronic spinal cord injury [58]. Taking into account the results of the above-mentioned studies, it seems to be possible that also in the pig, SP and PACAP may be involved in the regulation of the urinary bladder functions at different levels of neural control of this organ.

Our findings showed that BTX did not change the number of SP+/PACAP+ BPSN. As discussed earlier, it was revealed that PACAP [53] is upregulated during bladder cystitis and that this neuropeptide plays a very important anti-inflammatory function [57,59]. Therefore, on one hand the present results may suggest that microinjections of BTX into urinary bladder wall have not evoked any inflammatory responses, and, on the other hand, they confirm the results of previous studies reporting an anti-inflammatory effect of the BTX itself [49].

In the present experiment it was found that almost 36% of the total subpopulation of SP+ BPSN simultaneously contained CGRP. The results of the previous studies suggest that CGRP, which *per se* has no excitatory effect on the vesico-vesical reflex pathways [60], is able to facilitate the SP-evoked chemo-nociceptive reflex. CGRP acts synergistically with SP in the spinal cord [61]. The cooperation between CGRP and SP may result from the CGRP-mediated inhibition of an endopeptidase that degrades SP [62], which subsequently leads to rise in the local concentration of SP at the site of its release. It has also been shown that cystitis causes a significant increase in the number of SP- or CGRP-IR bladder-projecting cells in rat DRG [33,34]. These findings may suggest that CGRP can be indirectly involved in sensitization of afferent neuronal pathways in the lower urinary tract by enhancing SP action at the spinal cord level what, in turn, increases the urinary bladder excitability and pain sensation. It is also possible that CGRP somehow participates in pathogenesis of bladder overactivity. Moreover, it has been found that the release of CGRP from afferent nerve terminals increases in an acetic acid induced bladder pain model in rats [26], leading to bladder hyperactivity and pain sensation from this organ. Furthermore, Gamse and Saria [63] have shown in rats, that CGRP potentates SP inflammatory function. Thus, taking into account the above mentioned data it may be suggested that CGRP which is coexpressed with SP in the same BPSN may probably potentate the excitatory effect of SP, leading to an increase in bladder contractions, pain transmission, and inflammatory responses under pathological conditions both at the level of the spinal cord and the peripheral nervous system.

The present study has revealed that BTX injections into the urinary bladder wall cause a distinct decrease in the number of SP-positive DRG BPSN immunoreactive to CGRP. This observation corresponds well with findings of other authors obtained in experiments performed in rats. For example, it has been found that BTX may dramatically reduce both SP and CGRP release from capsaicin-sensitive sensory nerve endings projecting to the urinary bladder wall [26,49,64] and from the superficial bladder layers [65]. Moreover, it has been reported that BTX reduces a calcium-dependent and potassium-evoked release of SP from rat DRG neurons in embryonic primary culture system [9] and CGRP from cultured embryonic trigeminal ganglion neurons [10]. Furthermore, *in vivo* studies have demonstrated that the intrathecal delivery of BTX produces a prominent block of the evoked release of SP from small primary afferents [66]. The reduction in the number of SP/CGRP-IR bladder projecting primary afferent neurons after BTX bladder injections possibly weakens the excitatory effect of SP and CGRP on the preganglionic neurons in parasympathetic nuclei. This, in turn, may lead to an inhibition of bladder contractions and pain transmission under pathological conditions.

In the present study for the first time we provided evidence that the third most numerous subpopulation of SP-IR BPSN was that consisting of neurons simultaneously containing GAL. Moreover, our observations revealed that BTX treatment distinctly increased the number of SP+/GAL+ DRG sensory neurons supplying the urinary bladder wall.

Experiments performed on various animal models of neuropathic pain revealed both an increased synthesis of GAL in DRG and increased release of this neuropeptide from superficial layers of the spinal dorsal horn, leading to attenuation of pain transmission [67]. This anti-nociceptive effect is probably caused by activation of the inhibitory GAL1 receptors on putatively glutamate-containing excitatory dorsal horn neurons [68]. In the rat model of spinal cord injury it was observed that GAL may counteract the action of PACAP and nNOS on bladder-projecting afferent cells [69]. Callsen-Cencic and Mense [34] revealed that GAL inhibits presynaptically the release of SP and CGRP from bladder capsaicin-sensitive primary afferents following mustard oil-induced inflammation of the urinary bladder. Furthermore, it was found, that GAL antagonizes the facilitatory effect of SP on the nociceptive flexor reflex [70] and plays a tonic inhibitory role in mediation of spinal cord excitability. The aforementioned data suggest the modulatory action of GAL exerted on neural circuits participating in the regulation of urinary bladder function and on the transmission of pain from this organ. Therefore, the results of our study may suggest that BTX, by increasing GAL expression in SP-IR BPSN, can cause a decrease in SP release from afferent central terminals within the sacral spinal cord what leads to the reduction of its excitatory effect on the preganglionic neurons forming parasympathetic nucleus. Furthermore, it may be assumed that upregulation of GAL may cause an inhibition of bladder contractions and pain transmission under pathological conditions.

The present study has revealed that a small number of SP-positive DRG BPSN were immunoreactive to nNOS. NO is well known to act as a “retrograde transmitter” in afferent nerve fibers. Moreover it is involved in pain transmission in multisynaptic local circuits of the spinal cord [71]. It has been suggested that in the lower urinary tract NO plays an important role in the facilitation of the micturition reflex evoked by noxious chemical irritation of the bladder [72]. Furthermore, it is well documented that NO released from the afferent nerve terminals of the urinary bladder in rats participates in the initiation of inflammatory responses and is involved in triggering the painful sensations [73,74]. Basing on the above-mentioned data, it seems probable that also in the pig, SP and nNOS may alter transmission in neurons constituting the pathways regulating urinary bladder functions. However, the physiological relevance, the exact mechanism(s) and place(s) of action(s) of these neuropeptides, and their simultaneous functional interactions in the same neuron in the domestic pig remains to be addressed in detail.

In the present experiment, single FB+/SP+ DRG neurons were found to be immunopositive to SOM or CB. Moreover, BTX application was followed by a distinct decrease in the expression of these two neurotransmitters in the investigated nerve cells. Therefore it can be assumed that the therapeutic effect of BTX on the urinary bladder wall is at least partially mediated by down-regulation of the expression of SOM or CB in SP-positive BPSN. The simultaneous roles of SP and SOM or SP and CB in the regulation of urinary bladder functions, as well as the physiological relevance of a distinct decrease in the number of neurons forming these cell subpopulations after BTX treatment, are not clear. However, the available data suggest that SOM released into the spinal dorsal horn by peripheral nociceptive stimulation depresses the firing of dorsal horn neurons activated by noxious stimulation [75]. It has also been shown that SOM exerts a systemic antinociceptive effect [76] and inhibits acute neurogenic and non-neurogenic inflammatory reactions [77]. On the other hand, the main physiological relevance of CB in neurons is to act as a Ca^2+^ buffer, thus to control the Ca^2+^ level within the cytoplasm. It has been reported that CB not only functions as a passive Ca^2+^ buffer but also has an active role in neuronal activity [78]. It has been suggested that CB may be involved in some aspects of pain transmission, at least in small-sized SP-IR spinal ganglion neurons [79]. As was already mentioned, in porcine DRG the number of bladder sensory neurons containing simultaneously SP and CB distinctly decreased after BTX injections. Therefore it may be assumed, that the toxin administration may be followed by changes in Ca^2+^ homeostasis, which in turn, could result in a decrease in SP release from central afferent branches in the dorsal horn. This suppression of SP release would, on the other hand, diminish the urinary bladder excitability and pain transmission.

## 4. Experimental Section

### 4.1. Laboratory Animals

The study was performed on twelve juvenile (8–12 weeks old, 15–20 kg body weight—b.w.) female pigs of the Large White Polish race. The animals were kept under standard laboratory conditions. They were fed standard fodder (Grower Plus, Wipasz, Wadąg, Poland) and had free access to water. Before performing any surgical procedure, all the pigs were pretreated with atropine (Polfa, Poland, 0.04 mg/kg b.w., s.c.) and azaperone (Stresnil, Janssen Pharmaceutica, Beerse, Belgium, 0.5 mg/kg b.w., i.m.), and after thirty minutes the main anesthetic drug, sodium pentobarbital (Tiopental, 0.5 g per animal), was given intravenously in a slow, fractionated infusion. The depth of anesthesia was monitored by testing the corneal reflex. The animals were housed and treated in accordance with the rules of the local Ethics Commission (affiliated to the National Ethics Commission for Animal Experimentation, Polish Ministry of Science and Higher Education). All efforts were made to minimize the number of animals used and their suffering.

### 4.2. Surgical Procedures

In all the pigs, a mid-line laparotomy was performed and the urinary bladder was gently exposed to administer a total volume of 40 µL of 5% aqueous solution of the fluorescent retrograde tracer FB (K. Illing KG & Co GmbH, Gross Umstadt, Germany) into the wall of its right side in multiple injections with a Hamilton microsyringe equipped with a 26S gauge needle. To avoid leakage, the needle was left in each place of FB injection for about one minute. Three weeks later, which is an optimal time for the retrograde tracer to be transported to the DRG and labeled BPSN [41,80], the pigs were divided into two groups. Six pigs served as the controls and they were treated with multiple bladder injections of 5% aqueous solution of ethyl alcohol. Another group of six pigs was injected (multiple injections) into the right side of the urinary bladder wall with BTX type A (Botox^®^, Mayo, Ireland; 100 IU; 40 µL) using a cystoscope in order to mimic the route of its administration used in humans. One week after the administration of aqueous solution of ethyl alcohol or BTX, all the pigs were deeply anaesthetized with sodium pentobarbital and, after the cessation of breathing, transcardially perfused with freshly prepared 4% paraformaldehyde in 0.1 M (molar) phosphate buffer (pH 7.4). Bilateral DRG ganglia [41], together with the spinal cord were collected from all animals studied. Tissue samples were then postfixed in the same fixative (10 min at room temperature), washed several times in 0.1 M phosphate buffer and stored in 18% buffered sucrose at 4 °C until sectioning.

### 4.3. Sectioning of the Ganglia and Estimation of the Total Number of the DRG—BPSN

All the DRG studied were cut with a HM525 Zeiss cryostat on 10-µm-thick serial sections and examined using an Olympus BX51 fluorescence microscope equipped with an appropriate filter set for finding the FB+ neurons. To calculate the number of FB-positive perikarya, they were counted in every fourth section to avoid double counting of the same neuron (most neurons had approximately 40 µm in a diameter). Only neurons with clearly visible nucleus were considered. The total number of FB^+^ cells counted in all DRG from particular animal as well as the relative frequencies of perikarya in ganglia belonging to the particular neuronal classes were pooled and presented as mean ± SD. The diameter of the FB+ perikarya was measured by means of an image analysis software (version 3.02, Soft Imaging System, Münster, Germany).

### 4.4. Immunohistochemical Procedure

Immunohistochemistry involved double stainings which were performed according to an earlier described method [30]. They were applied to cryostat sections from both the ipsi- and contralateral DRG where the BPSN were found. Immunohistochemical characteristics of FB+ neurons were investigated using primary antibodies against biologically active substances including SP (rat monoclonal, AbD Serotec, Oxford, UK; 1:300), CGRP (rabbit polyclonal, Merck Millipore, Temecula, CA, USA; 1:9000), SOM (rat monoclonal, Merck Millipore, Temecula, CA, USA; 1:40), GAL (rabbit polyclonal, Merck Millipore, Temecula, CA, USA; 1:2000), PACAP (rabbit polyclonal, Peninsula, San Carlos, CA, USA; 1:15000), nNOS (mouse monoclonal, Sigma, St. Louis, MO, USA; 1:400) and CB (rabbit polyclonal, Merck Millipore, Temecula, CA, USA; 1:9000). The application of primary antisera raised in different species allowed to assess the coexistence of investigated biological active substances. Next, these primary antisera were visualized by rat- and mouse-specific secondary antisera conjugated to fluorescein isothiocyanate (FITC) or rabbit-specific antibodies conjugated to biotin (all from Jackson Immunochemicals, West Grove, PA, USA). The latter antibodies were then visualized by a streptavidin-CY3 complex (Jackson Immunochemicals, West Grove, PA, USA).

Retrogradely labelled, double-immunostained perikarya were evaluated under an Olympus BX61 microscope (Olympus, Hamburg, Germany) equipped with epifluorescence filter (Olympus, Hamburg, Germany) and an appropriate filter set for CY3 and FITC, and counted, in every fourth section (only neurons with clearly visible nucleus were included) in all the ganglia of all the animals. Relationships between immunohistochemical staining and FB distribution were examined directly by interchanging the filters. The colocalization of SP with other biologically active substances in FB+ neurons was examined on the consecutive sections obtained from all DRG studied. The percentages of the retrogradely labeled neurons immunopositive to particular biologically active substances or their markers investigated were pooled in all animals studied and presented as means ± SD.

Micrographs were taken using an Olympus XM10 digital camera (Olympus, Hamburg, Germany). The microscope was equipped with cellSens Dimension Image Processing software (Olympus, Hamburg, Germany). Morphometric data relative to each neuronal class were compared within each animal and among the animals, and were analyzed by the Student *t*-test using GraphPad PRISM 5.0 software (GraphPad Software, La Jolla, CA, USA). The differences were considered to be significant at *p* <0.05.

### 4.5. Control of Specificity of the Tracer Staining and Immunohistochemical Procedures

Thorough macroscopic examinations of the sites of FB injections and the tissues adjacent to the urinary bladder were performed before collecting the ganglia. The sites of the injections were easily identified by the yellow-labeled deposition left by the tracer within the bladder wall. Moreover, the injection sites were also observed in the UV lamp rays in the dark room. The tissues adjacent to the bladder were not found to be contaminated with the tracer. To verify that the tracer had not migrated into the urethra, we analyzed, in cryostat sections and by means of the H&E staining technique (IHC WORLD LLC, Woodstock, CT, USA), possible signs of leakage of the tracer to the junction between the urinary bladder trigone and cranial portion of the urethra. In all the animals studied no contamination of the tracer was found within the urethra. All these procedures excluded any leakage of the tracer and validated the specificity of the tracing protocol.

Standard controls, *i.e.*, preabsorption for the neuropeptide antisera (20 μg of appropriate antigen per 1 mL of corresponding antibody at working dilution; all antigens purchased from Peninsula, Sigma or Dianova, San Carlos, CA, USA, St. Louis, MO, USA or Hamburg, Germany, respectively), as well as omission and replacement of the respective primary antiserum with the corresponding non-immune serum completely abolished immunofluorescence and eliminated specific stainings.

## 5. Conclusions

The present study clearly revealed that BTX profoundly influences the chemical coding of SP-positive DRG nerve cells supplying the porcine urinary bladder wall. The changes after BTX treatment include a decrease in the number of SP+ BPSN containing simultaneously CGRP, SOM, or CB and an increase in the number of SP+ perikarya immunopositive to GAL. These observations suggest that alterations in the immunohistochemical properties of the sensory bladder innervation have to be taken into account when this neuroactive agent is used in the therapies of selected neurogenic urinary bladder disorders.
